# Implementation and validation of a beam‐current transformer on a medical pulsed electron beam LINAC for FLASH‐RT beam monitoring

**DOI:** 10.1002/acm2.13433

**Published:** 2021-10-05

**Authors:** Roxane Oesterle, Patrik Gonçalves Jorge, Veljko Grilj, Jean Bourhis, Marie‐Catherine Vozenin, Jean‐François Germond, François Bochud, Claude Bailat, Raphaël Moeckli

**Affiliations:** ^1^ Institute of Radiation Physics Lausanne University Hospital and Lausanne University Lausanne Switzerland; ^2^ Radiation Oncology Department Lausanne University Hospital and Lausanne University Lausanne Switzerland

**Keywords:** beam current monitoring, FLASH RT, ultra‐high dose rate

## Abstract

**Purpose:**

To implement and validate a beam current transformer as a passive monitoring device on a pulsed electron beam medical linear accelerator (LINAC) for ultra‐high dose rate (UHDR) irradiations in the operational range of at least 3 Gy to improve dosimetric procedures currently in use for FLASH radiotherapy (FLASH‐RT) studies.

**Methods:**

Two beam current transformers (BCTs) were placed at the exit of a medical LINAC capable of UHDR irradiations. The BCTs were validated as monitoring devices by verifying beam parameters consistency between nominal values and measured values, determining the relationship between the charge measured and the absorbed dose, and checking the short‐ and long‐term stability of the charge‐absorbed dose ratio.

**Results:**

The beam parameters measured by the BCTs coincide with the nominal values. The charge‐dose relationship was found to be linear and independent of pulse width and frequency. Short‐ and long‐term stabilities were measured to be within acceptable limits.

**Conclusions:**

The BCTs were implemented and validated on a pulsed electron beam medical LINAC, thus improving current dosimetric procedures and allowing for a more complete analysis of beam characteristics. BCTs were shown to be a valid method for beam monitoring for UHDR (and therefore FLASH) experiments.

## 1 INTRODUCTION

FLASH radiotherapy (FLASH‐RT) is based on the biological FLASH effect where for certain ultra‐high dose rate (UHDR) irradiations, there is an increase in differential response between tumor and healthy tissues.^1^ The exact mechanism and the triggers behind the FLASH effect are not yet fully understood and different explanations are proposed (see, e.g., refs. 2, 3, 4, 5, 6, 7, 8). Nevertheless, the advantage of the FLASH effect was already seen on multiple animal models, where healthy tissue was shown to be better protected in FLASH conditions than in conventional conditions. This led to the treatment of the first patient with FLASH‐RT.^9^, ^10^, ^11^, ^12^, ^13^, ^14^, ^15^, ^16^, ^17^

Despite its promising potential, one crucial problem emerging from using UHDR irradiations for clinical transfer is the need for new beam monitoring devices.^10^ This need comes from the heavy saturation that conventional transmission chambers usually used in clinical practice experience in UHDR modes. Although preliminary results show that a linear relationship can be found between dose and monitoring units (MU) for UHDR modes^18^ and a modified ionization chamber could be used as a potential monitoring device,^19^ another approach is needed due to the lack of intra‐pulse monitoring and the lack of data done with beams with larger field sizes. Using beam current transformers (BCTs), the beam is monitored in real time without beam perturbation and without saturation effects. An additional potential interesting advantage of using BCTs as UHDR beam monitoring instead of transmission chambers include the ability to check that the nominal values of the beam parameters (number of pulse, pulse width, pulse repetition frequency) coincide with the actual parameters read by the BCTs, allowing for an independent record and verification of the beam parameters used for the irradiation. Finally, the existence of a relationship between the measured current or charge and the absorbed dose, which could be used to define the actual delivered dose, is also a potential advantage. This is analogous to conventional LINACs, where the relationship is based on the current or charge produced in transmission chambers, leading to the definition of MU.

At the moment, a redundant dosimetric procedure developed specifically for UHDR is being used for beam monitoring in FLASH.^20^ A new and less time‐consuming beam monitoring based on BCTs implemented on electron beam LINACs was proposed and then its functionality was proven on the Oriatron eRT6 (PMB‐Alcen, France) prototype.^21^ The use of BCT as a monitoring device (and linked to interlocks) is already common in particle physics (e.g., CERN).

The aim of this work was to further check the validity of the use of BCTs as a passive beam monitoring device on a clinical UHDR electron beam LINAC in the UHDR operational range of at least 3 Gy, which is done by verifying beam parameter consistency, developing a charge‐absorbed dose relationship, and checking short‐ and long‐term stabilities.

## 2 MATERIALS AND METHODS

### 2.1 Medical device

The Mobetron (IntraOp, Sunnyvale, CA, USA) is a commercial medical LINAC used for intra‐operative radiotherapy and dermatology. It delivers pulsed electron beams from 6 to 12 MeV (hereafter called CONV mode). The device used in this study was modified to be able to deliver UHDR beams. Its possible configurations were one CONV mode at 9 MeV and two UHDR modes at 6 MeV and 9 MeV (nominal energies). In CONV mode, the pulse width (PW) and pulse repetition frequency (PRF) were fixed at 1.2 μs and 30 Hz respectively. In UHDR modes, the user could set the desired number of pulses between 1 and 200, the PW between 0.5 and 4 μs, and the PRF between 5 and 90 Hz. The commissioning of the modified Mobetron for FLASH‐RT preclinical biological experiments as well as FLASH‐RT clinical human protocols is described elsewhere.^22^

### 2.2 Dosimetric systems

**FIGURE 1:**
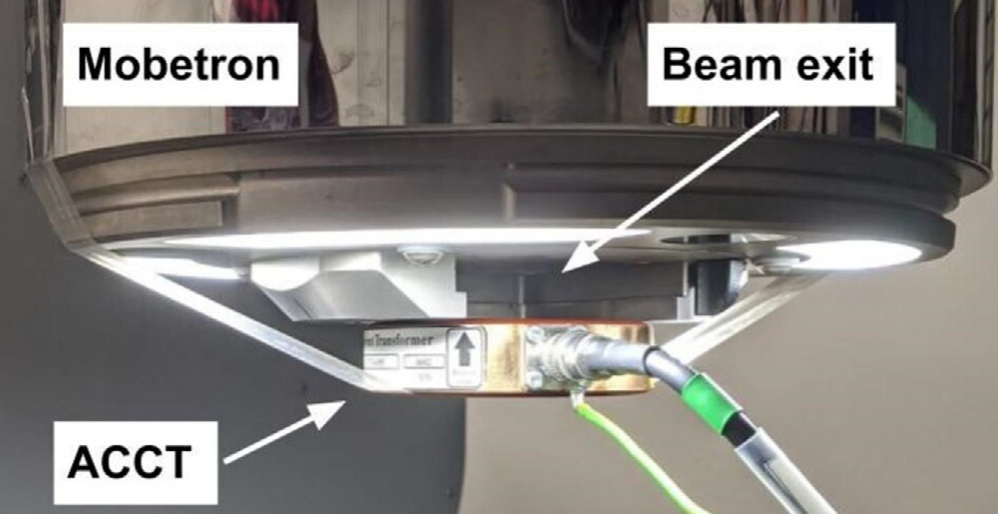
The setup used for measurements. The ACCT is centered and taped flat against the end of the head of the Mobetron. There is no direct contact between the ACCT and the primary collimator.

The BCTs used on the Mobetron are AC current trasnsformers (ACCTs) from Bergoz (Bergoz Instrumentation, Saint‐Genis‐Pouilly, France), which are toroid sensors connected to their own power supply and external electronic system.^23^ ACCTs measure the induced current of the electrons passing through them, thus giving a live and non‐destructive temporal readout of the beam. Two ACCTs were used, one with a full‐scale range of 10 mA (used for CONV irradiations) and the other 300 mA (used for UHDR irradiations), both with a rise time of 113 ns, a bandwidth of 3 MHz, signal drop of about 0.4% per millisecond, and an inner diameter of 55 mm.^23^ The ACCTs were implemented at the exit of the Mobetron, as seen in Figure 1. Beam profiles were done at this position to verify that the ACCT interferes minimally with the beam. As long as the beam passes through the inner circle of the ACCT, the value read is the same regardless of how centered the beam is. In the setup used in this paper, however, it is important that the ACCT is well centered as the beam size at the exit of the Mobetron leaves little margin in the inner circle of the ACCT.

The signal from the ACCT was acquired with a PXIe‐1071 oscilloscope with a NI PXIe‐5114 card (National Instruments, Austin, TX, USA) and then displayed and recorded with a LabVIEW 2014 VI software (National Instruments, Austin, TX, USA). After the acquisition, signal processing was done by applying a 20 MHz low‐pass filter, to remove random high‐frequency noise,^21^ followed by a baseline correction shift. The signal was then converted from voltage to current using the given Bergoz calibration coefficients.^23^ The sampling frequency could be selected on the LabVIEW interface and was chosen to be 125 MHz, as this frequency was verified to be high enough to not noticeably affect the pulse shape nor the total charge measured. The rising edge of the pulse was used as the trigger of the signal acquisition and was set at 5.5 μs. The first 5 μs were averaged and used for the baseline correction shift. The total net charge was calculated by integrating the filtered current signal with respect to time. The number of triggers during the acquisition gave the total number of delivered pulses. The PW was first measured as the distance between the 50% reference level instants (typical PW definition, according to the IEEE standard^24^) and as the distance between the 10% reference level instants (defines the start of the rising edge, according to the IEEE standard). As the 10% reference level PW corresponded more to the nominal PW, this definition was used for the rest of the paper. The PRF was determined by measuring the time between consecutive triggers. The LabVIEW VI interface calculated and displayed all of the aforementioned parameters along with the live readout of the pulse as voltage and the filtered pulse as current, both in function of time. This live readout showed the pulse shape of each pulse. The record of beam parameters for the typical beam temporal structure used in clinical LINACs is possible due to the bandwidth of the ACCTs, which is 3 MHz, and their short rise times.

In order to relate the ACCT signal to dose in a given condition, a charge‐dose relationship was measured in all modes. In the CONV mode, the dose was measured with an Advanced Markus ionization chamber from PTW (PTW‐Freiburg, GmbH, Freiburg, Germany) connected to an Unidos electrometer (PTW‐Freiburg, GmbH, Freiburg, Germany). For UHDR modes, EBT3 GafChromic films (Ashland Specialty Ingredients G.P., Bridgewater, NJ, USA), which have been proven to be efficient in UHDR mode due to a dose range of 0.1‐20 Gy and a 2% uncertainty independent of dose rate,^25^ were used for the charge‐dose measurements. Film scanning was done on an Epson V800 flatbed scanner (Epson, USA) and their calibration is described elsewhere.^26^

The Advanced Markus chamber was also used for the stability checks in all modes. The issue arising from chamber saturation^27^ in UHDR modes (as well as any polarization/recombination correction factors) can be neglected as only a relative comparison was done for these measurements. The dose can be determined with the Advanced Markus chamber with an uncertainty of 1.6% in CONV mode and 2.8% with the saturation model in UHDR modes,^27^ supporting our use of the chamber as a reference for the stability checks. The chamber should have enough time to reset itself between every pulse for all the frequencies used in this paper.

### 2.3 Beam parameters

The comparison of the nominal beam parameters (number of pulses, PW, and PRF) with the respective parameters read by the ACCTs was done for all modes.

The CONV mode on the Mobetron has a fixed PW of 1.2 μs and a PRF of 30 Hz. Therefore, in order to check the beam parameters of this mode, ten measurements for 180 pulses were done and the PW, PRF, and number of pulses read by the ACCT were averaged and compared to the nominal values.

For each UHDR energy, one measurement of two pulses in each possible configuration of PW and PRF was done (10 PW × 8 PRF = 80 configurations per energy). The parameters read by the LabVIEW were recorded for each measurement and compared with the nominal parameters. To get the average PW read by the ACCT, the ten individual PW were averaged over each PRF. Likewise, to get the average PRF, the eight individual values are averaged over each PW. The correspondence of the number of pulses was obtained with ten measurements of 100 pulses with a PW of 4 μs and a PRF of 60 Hz for each energy. The number of pulses read by the ACCT was then averaged and compared to the nominal value.

### 2.4 Charge‐dose relationship

The ACCT is of interest as a beam monitoring device if the charge read can be converted into dose. This conversion is created similarly to conventional methods, where the transmission chamber data is related to the absorbed dose to water in a reference setup.^28^ That relationship is then applied with information from commissioning, such as PDDs and output factors, to determine the corresponding dose in other conditions. For UHDR, that relationship is based on the ACCT charge instead of the transmission chamber current.

The reference setup chosen here was an open field at an SSD of 0.5 m and at 1 cm depth in solid water, which corresponds to 1–2 Gy per pulse for UHDR modes. For each measurement, one film was placed at this reference condition and the charge produced by the irradiation and read by the ACCT was recorded. The number of pulses (or the number of MU) given by the Mobetron was slowly increased in order to cover a wide range of charges/doses for UHDR modes (respectively CONV mode). The charge‐to‐dose relationship was compared for the full range of PW and PRF. Again, as the PW and PRF for 9 MeV CONV are fixed, only measurements for 1.2 μs and 30 Hz were done for this mode.

### 2.5 Short‐ and long‐term stabilities

In order to check the short‐ and long‐term stabilities of the charge‐dose ratios of the ACCTs, the Advanced Markus chamber was placed at the reference condition. Two pulses were given for the UHDR modes (4 μs, 60 Hz) and 100 MU for the CONV mode. Five measurements of the ratio of the Markus chamber charge (corrected for temperature and pressure) over the ACCT charge were done per day. These measurements were repeated for 8 days over the course of 3 months. The short‐term stability was calculated as the average standard deviation of the five daily ratios. The long‐term stability was calculated as the standard deviation of the daily averages of the ratios.

## 3 RESULTS

### 3.1 Pulse shapes

**FIGURE 2:**
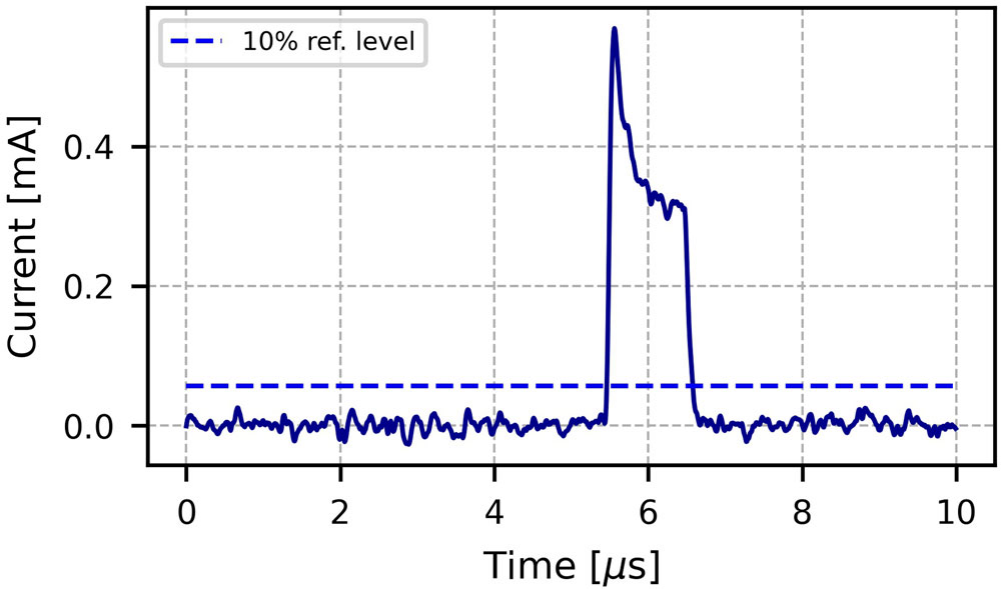
Pulse shape for 9 MeV in CONV mode. The blue dashed horizontal line shows the 10% reference level. The first intersection of this line with the pulse marks the start of the rising edge. The ACCT is triggered by the rising edge of the pulse. The first 5 μs are averaged and used for the baseline correction shift.

**FIGURE 3:**
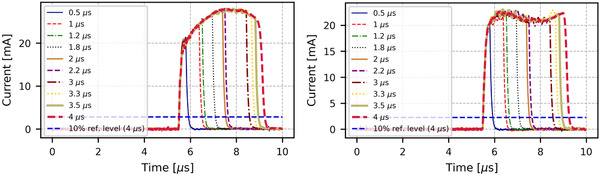
Pulse shapes for all PW at 6 MeV (left) and 9 MeV (right) in UHDR mode. The blue dashed horizontal line shows the 10% reference level for the 4 μs pulse. The first intersection of this line with the pulse marks the start of the rising edge. The ACCT is triggered by the rising edge of the pulse. The first 5 μs are averaged and used for the baseline correction shift.

The pulses as current in function of time recorded by the LabVIEW VI are presented in Figure 2 for 9 MeV CONV and in Figure 3 for 6 MeV and 9 MeV in UHDR mode.

### 3.2 Beam parameters

Table 1 shows the measured beam parameters of the CONV mode compared to the nominal ones.

**TABLE 1:** Nominal values versus measured values of various beam parameters for the CONV mode

	Nominal values	ACCT values
PW [μs]	1.2	1.14 ± 0.02
PRF [μs]	30	30.3 ± 0.1
Number of pulses []	180	179 ± 3

Abbreviations: ACCT, AC current transformer; PW, pulse width; PRF, pulse repetition frequency.

**FIGURE 4:**
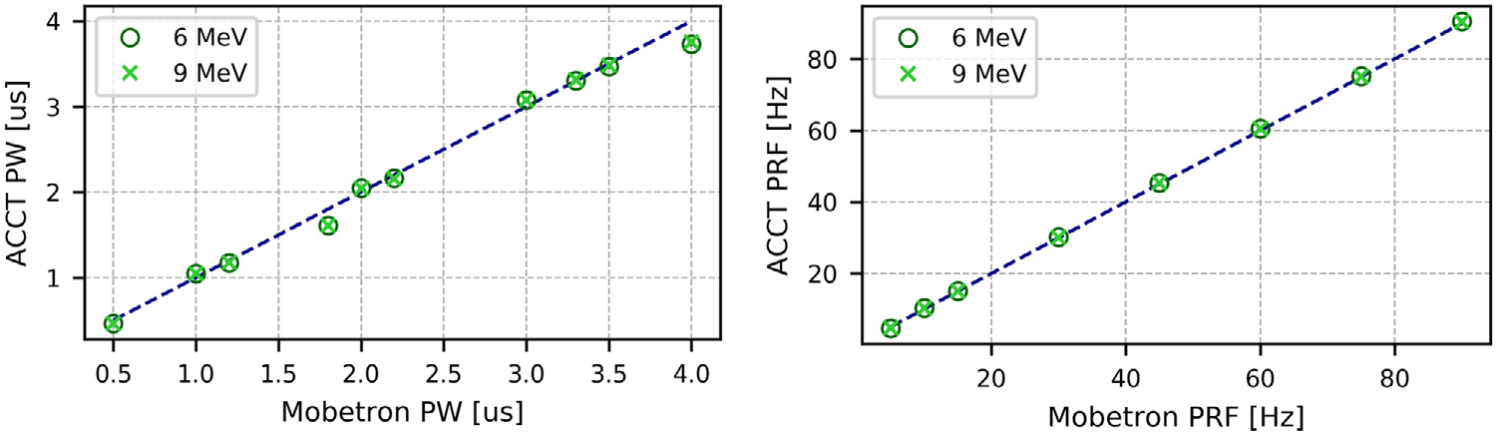
Measured versus nominal PW (left) and PRF (right) for UHDR mode. The blue dashed lines show where the ACCT values are equal to the Mobetron values. Error bars are smaller than the markers.

Figure 4 shows the PW and PRF of the UHDR modes. The average number of pulses was measured to be 100 ± 0 for 6 MeV UHDR and 99.6 ± 0.8 for 9 MeV UHDR. During the 10 measurements of 100 pulses for each energy, it was found that the PW does not vary for both energies and that the PRF did vary only by 0.3 Hz at 9 MeV UHDR (no variation at 6 MeV UHDR), implying that the beam parameters found in Table 1 are expected to hold true when a higher number of pulses is delivered.

### 3.3 Charge‐dose relationship

**FIGURE 5:**
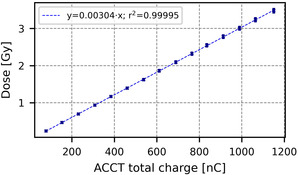
Charge‐dose relationship for 9 MeV in CONV mode

**FIGURE 6:**
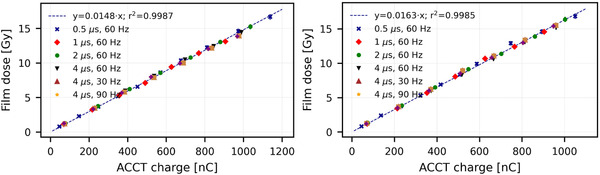
Charge‐dose relationship for 6 MeV (left) and 9 MeV (right) in UHDR mode

The charge‐dose relationship for the CONV mode is shown in Figure 5, where the dose (proportional to the Markus chamber charge) is plotted in function of the total ACCT charge. The average and maximal deviation of the expected dose from the actual dose are 0.36% and 0.8%, respectively. For UHDR, the points of each PW‐PRF configuration are combined in Figure 6 to get an overall trendline. The average deviations of the expected dose from the actual dose over the whole range of doses are 2.90% for 6 MeV and 2.51% for 9 MeV. The average and maximal deviations of the expected dose from the actual dose in the expected operational range (> 3 Gy) are respectively 1.62% and 4.10% for 6 MeV and 1.59% and 5.61% for 9 MeV. The average deviations for doses below the operational range are 10.8% for 6 MeV and 7.7% for 9 MeV. These higher values at lower dose are probably due to an increase in the uncertainty of the films (there is already a background level of ∼0.2 Gy) and not due to the ACCTs.

### 3.4 Short‐ and long‐term stabilities

The results of the stability check of the charge‐dose ratio are displayed in Table 2. The stability fluctuations are random (i.e., there is no overall shift in one direction or another).

**TABLE 2:** Results of the short‐ and long‐term stability check for the three energies. The short‐term stability refers to the average SD of the five daily ratios and the long‐term stability refers to the SD of the eight average daily ratios taken over the course of 3 months

	9 MeV CONV	6 MeV UHDR	9 MeV UHDR
Short‐term stability [%]	0.43	1.79	2.09
Long‐term stability [%]	2.38	2.85	3.98

Abbreviation: UHDR, ultra high dose rate.

## 4 DISCUSSION

The validity of the use of BCTs as a beam monitoring device on a clinical UHDR electron beam LINAC was checked by verifying beam parameter consistency, developing a charge‐absorbed dose relationship, and checking short‐ and long‐term stabilities.

The beam parameters measured by the ACCTs coincide suitably with the nominal values. A slight deviation from the nominal value was observed as the PW tends to 4 μs. This is due to physical limitation of the Mobetron's UHDR modes, as large PW and high PRF push the limit of the modulator. The charge‐dose relationship was linear and independent of PW and PRF. Even for noisy and low‐amplitude signals acquired in the CONV mode, the charge‐dose relationship for the Mobetron is remarkably linear. The differences from the fit show the possibility of an accurate estimation of dose. The short‐ and long‐term stabilities are both acceptable. The short‐term stability measured with the film is within the uncertainties of the dose measured by the film. The actual dose delivered can be estimated in the short‐term with a precision that depends on the short‐term stability and the difference from the fit. Combining these two uncertainties, the dose in the expected operational range (> 3 Gy) can be estimated with an average precision of 0.56% for CONV and 2.41% and 2.62% for 6 MeV and 9 MeV in short‐term. All of these individual points, along with the non‐destructive aspect of the ACCTs, support their validation as a beam monitoring device for UHDR electron beam irradiations. The drawback of using ACCTs is their inability to give information on beam shape and spatial distribution, as the only thing that is measured is the total flux of electrons going through the ACCT. Thus, certain operating parameters that can be monitored with conventional ionization chambers and are necessary for clinical use, such as beam energy, flatness, beam size, and symmery,^29^, ^30^ cannot be monitored with ACCTs.

At the moment, the only other LINAC on which ACCTs were implemented and validated is the Oriatron eRT6.^21^ The results of ACCTs on the eRT6 are compared to the results found with the Mobetron in Table 3. The biggest difference between both LINACs is the PW and PRF independence of the charge‐dose relationship for the Mobetron. This is advantageous when adjusting the final dose, because it is then possible to do it by slightly changing the PW, whereas it is not directly possible with the eRT6 because the charge‐dose relationship changes with PW. On the other hand, the stability of the charge‐dose ratio is slightly better with the Oriatron eRT6.

**TABLE 3:** Comparison of results of the validation of ACCTs on the Oriatron eRT6 versus the Mobetron

	Oriatron eRT6^21^		Mobetron	
	UHDR	CONV	UHDR	CONV
Consistent beam parameters?	Yes			
Short‐term stability [%]	1.4	0.5	1.8‐2.1	0.4
Long‐term stability [%]	1.5	0.9	2.8‐4	2.4
Charge‐dose relationship	Linear, dependent on PW		Linear, independent of PW	

Abbreviations: ACCT, AC current transformer; UHDR, ultra high dose rate.

Future integration of an ACCT in the head of the Mobetron would allow for easier use and for a more clinical environment. The next step in improving the use of ACCTs as beam monitoring would be to implement a beam‐stopping electronic‐based mechanism for total beam monitoring and a way to resolve the issues related to the inability to measure the beam spatial distribution. One possible solution for the latter could be to implement screens.

## 5 CONCLUSIONS

The use of ACCTs as a beam monitoring for UHDR on a medical device has been demonstrated. Various beam characteristics can be measured in real time. The additional advantage of being able to calculate the absorbed dose by using a single relationship depending on the measured charge, regardless of PW or PRF used, supports the use of ACCTs for UHDR electron LINACs.

## ACNOWLEDGMENTS

The study was partly supported by IntraOp Medical corporation, by Fondation pour le soutien de la recherche et du développement de l'oncologie (FSRDO), a Synergia grant from the FNS CRS II5_186369, a grant from ISREC Foundation thank to Biltema donation, a grant from the EMPIR program of the European Union Horizon 2020 research and innovation program, and a NIH program project grant PO1CA244091. The authors would like to acknowledge Derek Descioli, Kenneth Brooks, Sebastian Adamczyk, James Nelson, and Marc Swallyee of IntraOp Medical corporation for their technical support.

## AUTHOR CONTRIBUTION

All authors have made substantial contributions to conception and design, or acquisition of data, or analysis and interpretation of data, have been involved in drafting the manuscript or revising it critically for important intellectual content, have given final approval of the version to be published, have participated sufficiently in the work to take public responsibility for appropriate portions of the content and have agreed to be accountable for all aspects of the work in ensuring that questions related to the accuracy or integrity of any part of the work are appropriately investigated and resolved.

6

## CONFLICT OF INTEREST

The study was partially funded by IntraOp Medical corporation.

## Data Availability

The data that support the findings of this study are available from the corresponding author upon reasonable request.
