# Erratum: Implementation and validation of a beam‐current transformer on a medical pulsed electron beam LINAC for FLASH‐RT beam monitoring

**DOI:** 10.1002/acm2.13946

**Published:** 2023-02-27

Oesterle, R, Gonçalves Jorge, P, Grilj, V, et al. Implementation and validation of a beam‐current transformer on a medical pulsed electron beam LINAC for FLASH‐RT beam monitoring. *J Appl Clin Med Phys*. 2021;22(11):165–171. https://doi.org/10.1002/acm2.13433

In the acknowledgments, the citation for the EMPIR programme (…, a grant from the EMPIR programme co‐financed by the Participating States from the European Union's Horizon 2020 research and innovation programme, …) was missing the project number, the correct one is:

…the project 18HLT04 UHDpulse which received funding from the EMPIR programme co‐financed by the Participating States and from the European Union's Horizon 2020 research and innovation programme, …

In the acknowledgments, the citation for the EMPIR program was missing the project number as a reference.

We apologize for this error.

